# Examining Sociodemographic Factors, Reasons, and Barriers in the Diversity of Fruit and Vegetable Intake among Undergraduate Students

**DOI:** 10.3390/nu16060779

**Published:** 2024-03-09

**Authors:** Thanaporn Kaewpradup, Mutthatinee Tangmongkhonsuk, Charoonsri Chusak, Mario Siervo, Sirichai Adisakwattana

**Affiliations:** 1 Center of Excellence in Phytochemical and Functional Food for Clinical Nutrition, Department of Nutrition and Dietetics, Faculty of Allied Health Science, Chulalongkorn University, Bangkok 10330, Thailand; thanaporntk.t@gmail.com (T.K.); mutthatinee.t@gmail.com (M.T.); sirichai.a@chula.ac.th (S.A.); 2 Faculty of Health Sciences, School of Population Health, Curtin University, Perth, WA 6102, Australia; mario.siervo@curtin.edu.au; 3 Curtin Dementia Centre of Excellence, Enable Institute, Curtin University, Perth, WA 6102, Australia; 4 Vascular and Metabolic Disorders Group, Curtin Health Innovation Research Institute (CHIRI), Curtin University, Perth, WA 6102, Australia

**Keywords:** fruit and vegetable intake, undergraduate students, sociodemographic, reason, barrier

## Abstract

The transition from adolescence to university life represents a crucial period during which dietary choices can significantly influence long-term health outcomes. While the benefits of consuming a diet rich in fruits and vegetables (FVs) are widely acknowledged, there remains a noticeable gap in research concerning the factors influencing the consumption of specific FV varieties among university students. This study aimed to investigate the factors and barriers influencing the diversity of fruit and vegetable intake among undergraduate students. A cross-sectional study involving 542 undergraduate students (with an average age of 20.6 ± 0.1 years and a body mass index of 21.3 ± 0.2 kg/m^2^) was conducted at Chulalongkorn University in Bangkok, Thailand, between February and September 2022. Most students showed a preference for tropical fruits with inedible peels (88.2%) and *Brassicaceae* vegetables (91.0%), whereas lower consumption was observed for citrus fruits (19.7%) and *Fabaceae* vegetables (43.7%). Sociodemographic factors and cooking methods significantly influenced FV intake, with non-consumption associated with male students, independent living, lower BMI, and advanced academic years. A lower quality of life was found to be correlated with a higher proportion of students who did not consume vegetables. Barriers to inadequate fruit intake included busy lifestyles, while taste preference emerged as the primary reason for fruit consumption. Busy lifestyles and perceived healthiness were identified as the main barriers and reasons for vegetable intake. The study highlights the importance of implementing strategies and improvements in the university environment to promote diverse FV consumption and encourage healthy dietary behaviors among students.

## 1. Introduction

Fruits and vegetables are universally recognized as essential components of a healthy diet due to their rich nutritional profile, including dietary fiber, vitamins, minerals (notably electrolytes like potassium, calcium, and magnesium), and various phytochemicals that exhibit significant variation among different fruit and vegetable varieties [1]. These foods are crucial in mitigating risk factors associated with various chronic diseases, such as elevated blood sugar levels, lipid imbalances, and high blood pressure, while also countering oxidative stress [2,3,4].

The World Health Organization (WHO) recommends daily consumption of at least 400 g or five servings of fruits and vegetables to enhance overall health [5]. Several studies consistently highlight that increased fruit and vegetable intake correlates with reduced inflammation and lower susceptibility to chronic diseases, including diabetes, coronary heart disease, stroke, cancer, and all-cause mortality [6]. Evidence suggests that higher fruit and vegetable consumption is associated with a 7% to 27% reduction in the risk of cardiovascular disease (CVD), coronary heart disease (CHD), and stroke incidents [7]. A linear dose-response relationship exists between the intake of these foods and the risk of CHD, with a maximum daily consumption of seven servings of each fruit and vegetable leading to approximately 20% and 30% reductions in CHD incidence and mortality, respectively [4]. However, a substantial proportion of countries, representing 75% of the global population, fall below the WHO’s recommended minimum targets for fruit and vegetable intake [8].

Inadequate fruit and vegetable consumption is a complex issue influenced by diverse factors that vary across age groups and demographic categories. For example, surveys conducted in the United States and Australia highlight a concerning trend, with 82% of adults failing to meet the recommended daily vegetable intake due to factors such as attitudes, time constraints, and accessibility issues [9,10,11]. Despite extensive studies on consumption patterns that often focus on broad age ranges and general populations, there is a noticeable oversight in addressing specific data gaps, particularly those related to young adults. Understanding the dietary habits of this demographic is paramount, given that their lifestyle choices can significantly impact the risk and severity of chronic diseases later in life.

Targeting specific demographics, such as young adults, becomes imperative to gain insights into their consumption behaviors and associated factors regarding fruit and vegetable intake. This understanding lays the foundation for developing effective strategies to modify dietary behavior, promote increased fruit and vegetable intake, and mitigate future health risks. The issue of inadequate fruit and vegetable consumption is particularly pronounced among young adults, especially undergraduate students, who consistently face challenges in maintaining a balanced and nutritious diet, with low fruit and vegetable intake being a notable concern [12,13]. Additionally, a lack of awareness about the nutritional benefits of fruits and vegetables and their role in promoting health may contribute to their limited inclusion in the diet.

Notably, there is a significant gap in surveys regarding the varieties of fruit and vegetable consumption among undergraduate students. To address this gap, this study aimed to examine the intake of fruits and vegetables, analyze each variety independently, and identify the factors and barriers influencing such intake among undergraduate students. The goal was to comprehensively understand their eating behaviors, providing valuable insights for creating targeted strategies and practical interventions to promote increased fruit and vegetable consumption among this demographic.

## 2. Materials and Methods

### 2.1. Study Design and Participants

This online cross-sectional study was conducted from February to September 2022. The questionnaire was administered via Google Forms on an online platform and distributed through various social media channels, including email, Facebook, Instagram, and Line. Additionally, QR codes were generated for the questionnaire distribution to students onsite at locations such as canteens, dormitories, the sports center, and the university library. Information sheets and consent forms were provided to all participants.

The study included male and female undergraduate students from Chulalongkorn University aged ≥ 18 who were proficient in reading and understanding Thai. Participants with incomplete questionnaire responses were excluded from the analysis.

**Figure 1:**
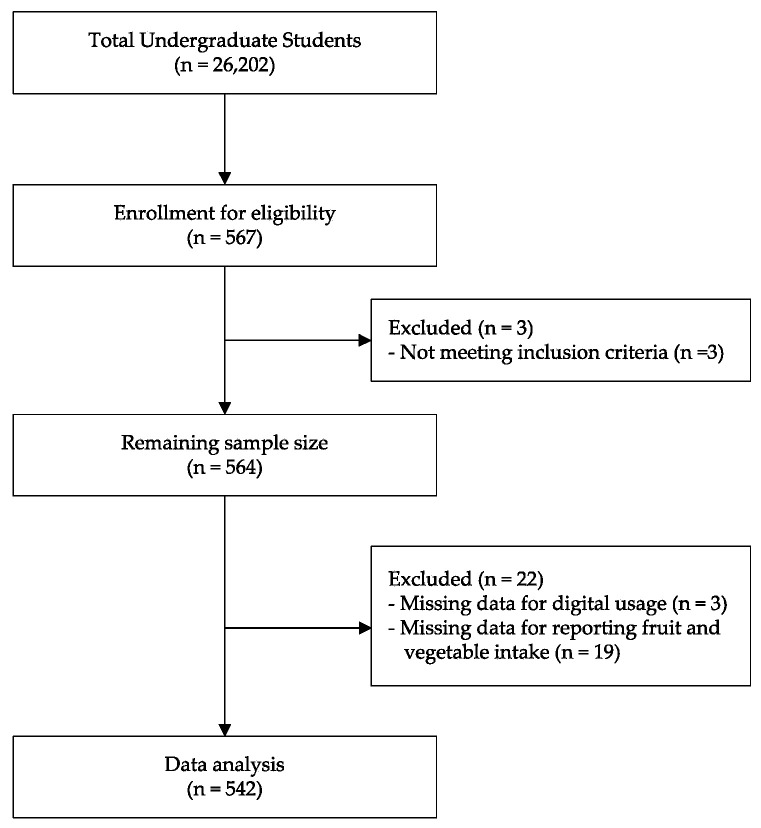
Consort diagram of study flow and participants.

The sample size was determined based on the undergraduate student population of Chulalongkorn University, calculated with a 5% margin of error at a 95% confidence level and accounting for a 20% dropout rate, suggesting that a minimum of 455 students should be included. The study protocol was conducted following the Declaration of Helsinki and approved by the Research Ethics Review Committee for Research Involving Human Subjects at Chulalongkorn University (COA No. 019/2565). The details of the study flow and participant enrollment are shown in Figure 1.

### 2.2. Data Collection

Data collection involved using an online questionnaire, which was promoted through email, Facebook, Instagram, and Line. Undergraduate students were invited to participate in a questionnaire focusing on the correlation between sociodemographics, stress, quality of life, and barriers to fruit and vegetable intake. Before completing the online questionnaire, the researcher provided participants with an overview of the study’s objectives and a brief description of the questionnaire.

### 2.3. Questionnaire Design

The questionnaire, structured as an online form, consisted of five sections: (1) General Information (14 questions), (2) Fruit and Vegetable Intake (8 questions), (3) Reasons and Barriers for Consumption (5 questions), (4) Stress (10 questions), and (5) Quality of Life (26 questions). Completing the entire questionnaire typically took approximately 10–15 min. It comprised a total of 63 questions, including blanks, rating scales, and multiple-choice options. Three experts in the fields of nutrition and public health evaluated each question for validity and reliability, with the overall instrument demonstrating reliability, as indicated by Cronbach’s alpha index of 0.95. In the Sociodemographic Characteristics section, participants provided background information, including sex, age, study fields, academic years, living arrangements, engagement in online learning, digital usage, physical activity, smoking habits, and cooking methods. Hybrid learning, a common practice in universities, was closely linked to the duration of digital usage, encompassing tablets, computers, and smartphones. Consequently, participants were queried about the duration of online learning and digital platform usage, classified based on the credit registration criteria for undergraduate students at Chulalongkorn University (≤3 h/>3 h/day for online learning, and <3 h/day, 3–6 h/day, or >6 h/day for digital usage). Lifestyle behaviors were assessed following the WHO guideline on physical activity (no exercise, <3 times or 150 min/week, or ≥3 times or 150 min/week), categorizing physical activity levels as inactivity, insufficient, and sufficient [14]. Additionally, participants indicated their cooking methods (by themselves, by parents or caregivers, or by buying food from outside) to evaluate their behavior regarding food preparation.For the assessment of fruit and vegetable intake, a semi-FFQ questionnaire was utilized. This questionnaire delved into the frequency and quantity of 24 fruits and 20 vegetables. Supplementary Table S1 represents common types consumed by Thai people and meeting Thai agricultural standards, categorized into six and seven families, respectively [15]. The supporting materials provided a list of the specific fruits and vegetables included in the questionnaire, as shown in Supplementary Figures S1 and S2. Additionally, participants were asked to specify the form in which they typically purchased fruits and vegetables (raw, cooked, or processed), and multiple answers were accepted.

Three response options “because of (taste/healthy/availability)” were provided to understand the reasons behind fruit and vegetable intake. In contrast, barriers to inadequate fruit and vegetable intake, aligned with the WHO recommendations, were assessed using options such as “because of (sufficient intake perceived/Unlike/It needs too much effort and time to prepare/I often forget to eat/It is expensive/It doesn’t fit my lifestyle/unknown benefits)”. Importantly, participants were allowed to select multiple answers for both sections [16]. Stress levels were gauged using the Perceived Stress Scale (PSS-10), using the previously validated Thai version [17]. Participants were asked to evaluate their stress over the past two weeks, with the total score ranging from 10 to 40 and categorized as 10 to 20 (low), 21 to 31 (moderate), and 32 to 40 (high). Additionally, the quality of life was assessed using the validated Thai version of the World Health Organization Quality of Life measurement form (WHOQOL-BREF-THAI) [18]. This assessment comprised 26 questions covering physical health, psychological health, social relationships, and environmental aspects related to quality of life. The total score ranged from 26 to 60 (poor) and 61 to 95 (mild), with a higher score indicating a higher quality of life.

### 2.4. Statistical Analysis

Data were represented as counts and percentages for categorical variables. The χ^2^ test was used to determine the relationship between all categorical variables. Binary logistic regression was used to assess the association between the consumption of each fruit and vegetable group (Yes/No intake) and independent variables (sociodemographic variables, physical activity level, cooking method, stress, and quality of life). For analysis, each subgroup of categorical variables including male, obese, a health science student, a senior student, living with parents, having an income THB > 10,000/month, attending online classes >3 days/week, using a digital device for online learning >6 h/day, engaging in sufficient physical activity, not smoking, cooking by themselves, reporting low perceived stress, and having poor quality of life were identified as a reference group. The adjusted odds ratio (OR) was applied with a 5% significance level, and statistical significance was considered at *p* < 0.05. All analyses were performed using the Statistical Package for the Social Sciences (SPSS) version 22.

## 3. Results

### 3.1. Sociodemographic Characteristics

A total of 542 undergraduate students participated in the study; 65.3% were female, and 29.0% were seniors (Table 1). Approximately 38% studied health sciences and lived in shared accommodation. More than half of the students had a normal BMI and received a monthly income of THB 5001–10,000. A total of 51.8% of students attended online classes more than 3 days per week, and 90.0% used digital devices for at least 3 h daily without smoking. Most students (40.0%) had insufficient physical activity. A minority reported high perceived stress (1.5%) and poor quality of life (2.4%). A majority of students (74.7%) purchased their food from outside rather than cooking by themselves or relying on family preparation.

### 3.2. Fruit and Vegetable Intake

**Figure 2:**
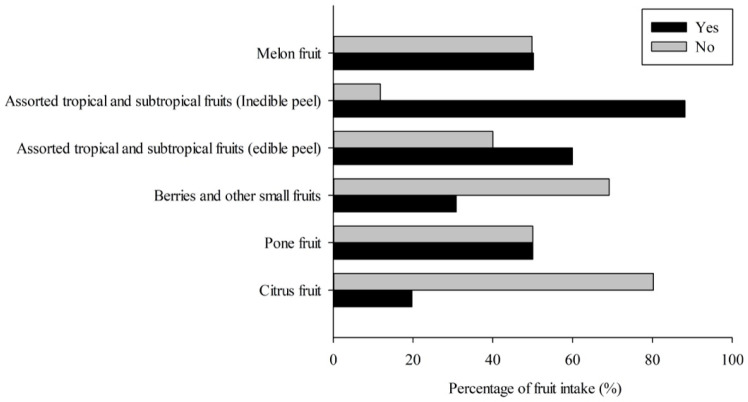
The percentage of fruit intake in undergraduate students.

**Figure 3:**
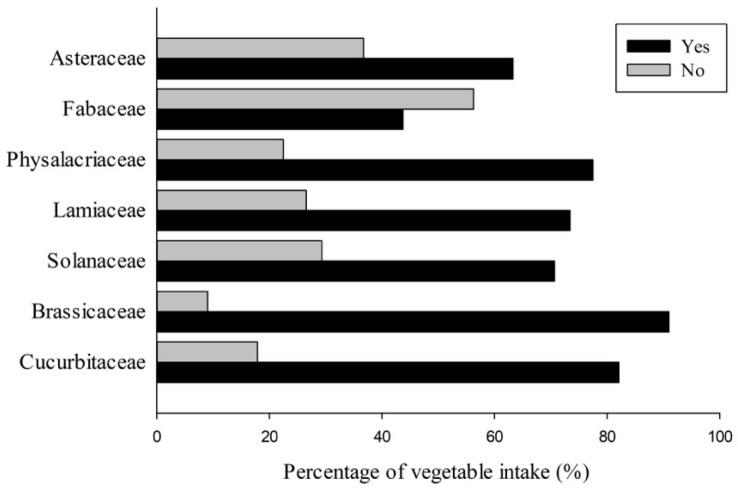
The percentage of vegetable intake in undergraduate students.

More than 90% of undergraduate students consumed less than three servings of fruit and less than four servings of vegetables per day (Table 1). The prevalence of students reporting their consumption or non-consumption of fruits and vegetables is depicted in Figure 2 and Figure 3. In terms of fruit intake, a higher percentage of students (80.3% and 69.2%) reported no intake of citrus fruit and berries/small fruit, respectively. Regarding vegetable intake, the family of *Fabaceae* vegetables had approximately 56.3% of students reporting no intake, which was higher than that of other vegetable families. Among the consumed items, inedible-peel-assorted tropical and subtropical fruits and *Brassicaceae* vegetables recorded the highest consumption rates (88.2% and 91%), while citrus fruits and *Fabaceae* vegetables had the lowest proportions (19.7% and 43.7%). Additionally, raw fruit and cooked vegetables were found to be the main purchased forms for both groups (Yes/No consumption), as shown in Supplementary Tables S2 and S3.

Supplementary Tables S4 and S5 present the correlation between sociodemographic factors and the variety of fruit and vegetable (FV) intake. Regarding fruit varieties, there was a significant association between gender and the intake of pone and edible peel of assorted tropical and subtropical fruits. Living arrangements were also significantly associated with the consumption of citrus fruits, pome, berries, and other small fruits and the inedible peels of assorted tropical and subtropical fruits. Additionally, the cooking methods used by students showed a significant association with the consumption of citrus fruits, berries, and other small fruits, as well as the inedible peels of assorted tropical and subtropical fruits. However, BMI, study field, academic years, income, online learning, digital usage, physical activity, smoking, stress, and quality of life did not significantly affect the decision to consume each type of fruit.

Concerning the diversity of vegetable consumption, significant associations were observed between gender and the intake of *Lamiaceae* vegetables. There was also a significant association between BMI and the consumption of vegetables from the *Physalacriaceae* and *Asteraceae* families. The field of study exhibited an association with the intake of *Physalacriaceae* vegetables, while different academic years were significantly associated with the consumption of *Brassicaceae* vegetables. Income was also found to be significantly associated with the consumption of *Asteraceae* vegetables. Moreover, a significant relationship was noted between the level of physical activity and the intake of *Solanaceae* and *Asteraceae* vegetables, respectively. Additionally, the choice of cooking methods was associated with the consumption of *Brassicaceae* and *Lamiaceae* vegetables. Lastly, the quality of life of students exhibited statistically significant associations with the intake of vegetables such as *Brassicaceae*, *Lamiaceae*, and *Physalacriaceae*, respectively. Nevertheless, the results did not show a significant association between the varieties of vegetable consumption and online learning, digital usage, smoking, or stress levels among undergraduate students.

Therefore, binary logistic regression was employed to examine the sociodemographic factors influencing the intake of various fruit and vegetable varieties. Regarding fruit consumption, the results from Table 2 revealed that female students were 1.6 and 2.2 times more likely than male students to consume pome fruit (95% CI = 1.086–2.410; *p* = 0.018) and the edible peel of assorted tropical and subtropical fruits (95% CI = 1.499–3.363; *p* ≤ 0.001). The odds ratio for female students reporting no consumption was 38.2% (95% CI = 0.415–0.921; *p* = 0.018) and 55.5% (95% CI = 0.297–0.667; *p* ≤ 0.001) lower for these fruits compared to male students.

Students who resided independently or with roommates demonstrated a notable correlation with the intake of pome fruits (less than 42.4% (95% CI = 0.342–0.969; *p* = 0.038) and 40.8% (95% CI = 0.370–0.946; *p* = 0.028), respectively) and berries and other small fruits (less than 62.5% (95% CI = 0.210–0.670; *p* ≤ 0.001) and 53.1% (95% CI = 0.283–0.776; *p* = 0.003), respectively) in comparison to those residing with their parents. In the no-intake group, students living alone or with roommates were 1.7 times (95% CI = 1.032–2.920; *p* = 0.038, 95% CI = 1.057–2.700; *p* = 0.028) more likely to abstain from pome fruits and 2.7 and 2.1 times (95% CI = 1.493–4.760; *p* ≤ 0.001, 95% CI = 1.288–3.532; *p* = 0.003) more likely to avoid berries and other small fruits than those living with their parents.

Furthermore, students who ingested meals prepared by their parents or purchased externally were 7.2 and 3.3 times (95% CI = 1.957–26.307; *p* = 0.003, 95% CI = 1.291–8.425; *p* = 0.013) more inclined than those who cooked for themselves to partake in the inedible peel of assorted tropical and subtropical fruits. Conversely, the odds ratio for students refraining from consuming these fruits was 86.1% (95% CI = 0.038–0.511; *p* = 0.003) and 69.7% (95% CI = 0.119–0.775; *p* = 0.013) lower among those who relied on food prepared by their parents or bought from outside, respectively, in comparison to those who engaged in self-cooking. Nevertheless, no statistically significant association was observed between the consumption of individual fruit varieties and other sociodemographic variables, as evidenced in Supplementary Table S6.

As depicted in Table 3, the odds ratio for students reporting *Lamiaceae* vegetable consumption was 66.7% (95% CI = 0.152–0.732; *p* = 0.006) lower among those with a normal BMI compared to obese students, while being three times more likely to have no consumption of these vegetables than obese students (95% CI = 1.366–6.593; *p* = 0.006). Concerning *Physalacriaceae* consumption, the odds ratio was significantly 64.8% (95% CI = 0.147–0.842; *p* = 0.019) and 74.6% (95% CI = 0.100–0.642; *p* = 0.004) lower among students with a normal BMI and underweight, respectively. In contrast, the odds ratio was 2.8 and 3.9 times higher among students with a normal BMI and underweight (95% CI = 1.188–6.79; *p* = 0.019, 95% CI = 1.557–9.953; *p* = 0.004), indicating a greater likelihood of no consumption of this vegetable compared to obese students.

Concerning the year of study, junior students were found to be 3.7 times more likely than senior students to report consuming *Brassicaceae* vegetables (95% CI = 1.423–9.414; *p* = 0.007), while the odds ratio for junior students reporting no consumption was 72.7% lower compared to senior students (95% CI = 0.106–0.703; *p* = 0.007). Additionally, students who consumed food prepared by their parents or bought from outside were significantly associated with reporting the consumption of *Brassicaceae* vegetables (3.7 and 3.5 times, respectively) (95% CI = 1.034–13.064; *p* = 0.044, 95% CI = 1.242–10.113; *p* = 0.018) and *Lamiaceae* vegetables (3.2 and 3.6 times, respectively) compared to those who cooked their food (95% CI = 1.268–8.053; *p* = 0.014, 95% CI = 1.578–8.253; *p* = 0.002). Conversely, students who consumed food prepared by their parents or bought from outside were significantly associated with reporting no consumption of *Brassicaceae* vegetables (less than 72.8% (95% CI = 0.077–0.967; *p* = 0.044) and 71.8% (95% CI = 0.099–0.805; *p* = 0.018), respectively) and *Lamiaceae* vegetables (less than 68.7% (95% CI = 0.124–0.789; *p* = 0.014) and 72.3% (95% CI = 0.121–0.634; *p* = 0.002), respectively) compared to those who prepared their meals.

Additionally, students with a mild and good quality of life were significantly associated with reporting higher consumption of *Brassicaceae* (12.7 and 9.8 times, respectively) (95% CI = 3.201–50.779; *p* ≤ 0.001, 95% CI = 2.330–41.079; *p* = 0.002), *Lamiaceae* (5.9 and 7.1 times, respectively) (95% CI = 1.645–21.266; *p* = 0.006, 95% CI = 1.900–26.298; *p* = 0.004), and *Physalacriaceae* (5.4 and 4.4 times, respectively) compared to those with a poor quality of life (95% CI = 1.548–18.905; *p* = 0.008, 95% CI = 1.215–15.733; *p* = 0.024). For students reporting no consumption of these vegetable families, the odds ratio was significantly lower—22.0% (95% CI = 0.020–0.312; *p* ≤ 0.001) and 89.8% (95% CI = 0.024–0.429; *p* = 0.002) for *Brassicaceae*, 83.1% (95% CI = 0.047–0.608; *p* = 0.006) and 85.9% (95% CI = 0.038–0.526; *p* = 0.004) for *Lamiaceae*, and 81.5% (95% CI = 0.053–0.646; *p* = 0.008) and 77.1% (95% CI = 0.064–0.823; *p* = 0.024), for *Physalacriaceae*, among those who consumed food prepared by their parents or bought from outside, respectively, compared to those who cooked for themselves. The regression results showed no statistically significant association between other sociodemographic characteristics and the consumption of each vegetable family, as indicated in Supplementary Table S7.

### 3.3. Reasons for Fruit and Vegetable Consumption

As indicated in Table 4, the percentage of students articulating their rationales for fruit and vegetable consumption displayed a consistent trend in both the cohort of students who incorporated them into their diet and those who abstained. Concerning fruit consumption, a dominant majority of students, 85.4%, accorded high priority to taste as a significant factor, whereas availability exhibited the least impact, acknowledged by only 42.4% of respondents. Regarding vegetable intake, 80.7% ascribed importance to health benefits, with availability registering the lowest impact at 23.3%.

### 3.4. Barriers to Fruit and Vegetable Consumption

The findings reveal that more than 50% of students exhibited insufficient fruit and vegetable intake, attributed to their hectic daily schedules (50.1% for fruit and 56.9% for vegetables). While approximately one-third of students perceived their fruit intake as adequate, a notable factor contributing to insufficient vegetable consumption was vegetable dislike, particularly among those reporting no intake of fruits and vegetables (30.4% for fruits and 77.8% for vegetables).

Among students who consumed fruits and vegetables, the primary factors contributing to inadequate fruit intake were a busy lifestyle (50.1%), already having sufficient intake (32.5%), and forgetfulness (29.2%). In contrast, the main factor for insufficient vegetable intake was a dislike of vegetables (34.6%). Additionally, about 1% of students reported being unaware of the benefits of consuming both fruits and vegetables (Table 5).

## 4. Discussion

Increasing daily fruit and vegetable consumption can mitigate cardiovascular disease risk factors and promote overall health [19,20]. However, limited research on young adults’ dietary habits regarding fruits and vegetables is limited. Our study aimed to understand the factors influencing fruit and vegetable consumption among undergraduate students. Our findings revealed that over 90% of undergraduate students had insufficient fruit and vegetable intake, consuming <3 servings of fruit and <4 servings of vegetables per day. The deficiency in fruit and vegetable intake largely indicates that their busy lifestyles are the primary obstacle, mainly due to the time commitments associated with attending classes [21,22]. Furthermore, the perception of already having sufficient intake serves as a significant barrier, leading to limited efforts to increase fruit and vegetable consumption within this demographic group [23].

Additionally, we found that female students consumed more pome and edible peel of assorted tropical and subtropical fruits compared to male students. These fruits are rich in phytochemicals and have a lower glycemic index (GI), aligning with the health and beauty consciousness often observed among women, who tend to favor lower-GI diets and phytonutrient-rich foods [24]. Conversely, males tended to consume more higher-GI fruits, prioritizing physique-related aspects and favoring diets rich in high-protein and energy-dense foods [24,25].

Living independently is associated with a lower intake of pome fruits, berries, and small fruits compared to students living with their parents. Living with family facilitates access to healthy food choices and promotes fruit preferences, possibly due to the presence of family members who can prepare a variety of fruits for students [26,27,28]. Conversely, students living independently tend to have less favorable dietary habits and lower fruit intake [29]. As previously reported, they frequently adopt unhealthy dietary patterns, including increased consumption of fast and processed foods, typically low in fruits [30].

For vegetable intake, *Brassicaceae* vegetables, known for their preventive health effects, contribute to lower blood pressure and enhanced cardiovascular well-being by increasing nitrate content in the body [31,32]. Despite their availability in Asian countries [33], our observations show that students who cook their meals consume fewer *Brassicaceae* vegetables compared to those whose meals are prepared by their families. Previous research aligns with our findings, indicating that decreasing consumption of these vegetables correlates with increased sensitivity to bitter tastes [34]. This discovery emphasizes the significance of taste preference as a key factor influencing students’ avoidance of vegetables. As a result, students may choose not to include these vegetables in their homemade dishes.

Moreover, prior studies have highlighted a parallel pattern to our findings, indicating that students in more advanced academic years exhibit reduced vegetable consumption, notably reporting a higher proportion of abstaining from *Brassicaceae* vegetables compared to those in earlier academic years [35]. Several barriers contribute to this pattern, including limited access, convenience challenges, and time constraints, which are more prevalent among students in higher academic years [36]. Additionally, academic advancement is often associated with increased perceived stress due to lifestyle demands, particularly academic workload [37]. Consistent with previous findings, higher academic levels are linked to lower frequencies of consuming healthy foods such as vegetables [38,39]. Moreover, the transition to a university environment can impact food sources, with a lack of healthy meal options at university canteens identified as a significant barrier to low vegetable consumption among students [21,40].

In addition to nutritional status, normal-weight or underweight students were significantly more likely to report no consumption of *Lamiaceae* and *Physalacriaceae* vegetables compared to their obese counterparts. This may reflect their differing levels of health consciousness, with obese students more inclined to include vegetables in their diets [41]. Meanwhile, self-esteem among young adults may play a role, as those with slimmer body shapes may feel less pressure to modify their diets for weight loss, resulting in lower motivation to consume nutritious foods [42,43]. Moreover, as shown in Supplementary Table S3, purchasing patterns indicate that a substantial proportion of students within a normal BMI range frequently purchase cooked vegetables.

Furthermore, we found a significant association between a decline in students’ quality of life (QOL) and reduced consumption of *Brassicaceae*, *Lamiaceae*, and *Physalacriaceae* vegetables. The transition after enrollment changes students’ behavior regarding greater responsibility for managing their lifestyles and acquiring knowledge to become professionals in their fields [44,45]. This transition is often accompanied by elevated stress levels, adversely affecting overall quality of life across various demographic groups [46,47]. External factors, such as disease pandemics, can also impact eating attitudes and dietary behaviors, leading to a preference for unhealthy diets and reduced vegetable intake [48,49,50].

To promote diverse fruit and vegetable consumption among university students, strategies could focus on expanding the variety of less-consumed fruit and vegetables in canteen menus [51,52]. Furthermore, establishing a health-conscious environment within the university, potentially through integrating artificial intelligence to develop more efficient nutritional tools, could boost fruit and vegetable consumption and foster healthier dietary practices among university students [53]. Education on nutritional knowledge and recommendations should start early in university life to enhance proper dietary attitudes and behaviors among students [54].

This study acknowledges limitations. It exclusively involved Thai undergraduate students from a capital university, limiting generalizability to students in other regions of Thailand. Fruit and vegetable intake assessment relied on self-reports, focusing on frequency and quantity. Incorporating dietary recall or food records could improve accuracy, reducing overestimation or underestimation of intake.

In addition, Chulalongkorn University, where the study was conducted, allows students from all regions in Thailand to be admitted. The difference in fruit and vegetable intake may also depend on the basic eating behaviors and lifestyles of individuals from various regions. Therefore, inquiring about the origin of the participants’ regions would enable a more effective exploration of fruit and vegetable intake among the students.

The chosen list of fruits and vegetables may not fully represent all types consumed by students. A more detailed list, coupled with an exploration of sociodemographic factors linked to each variety, would offer a nuanced view of students’ dietary patterns. Moreover, the illustrated portion sizes may not encompass all varieties, posing challenges in accurate estimation and introducing potential bias. Including a broader range of fruits and vegetables would enhance precision in portion size estimation in the study.

Despite these limitations, the study has several strengths. The implementation of an online questionnaire was cost-effective and facilitated efficient data collection. Categorizing fruit and vegetable types into major categories prevalent in Thailand provides valuable insights into the factors influencing a diverse range of fruit and vegetable consumption. Consequently, this dataset stands as a valuable representation of dietary patterns related to fruits and vegetables among undergraduate students.

## 5. Conclusions

The behaviors of young adults play a pivotal role in predicting their habits and health status in adulthood. The prevalence of inadequate fruit and vegetable intake remains notably high among university students and is positively linked to several contributing factors. The findings in this study underscore that specific fruit and vegetable types are less popular and less frequently consumed by undergraduate students, with varied associations between sociodemographic factors and the intake of different varieties. It is crucial to note that these factors may differ among students in various groups, such as those pursuing different study disciplines and residing in different regions. Therefore, gaining insights into the unique barriers and factors related to consumption within distinct study disciplines and regions may contribute to an increase in fruit and vegetable consumption and the promotion of healthier dietary behaviors among university students.

## Figures and Tables

**Table 1:** Sociodemographic characteristics of undergraduate students (*n* = 542).

Variables	Total, *n* (%)
Sex	
Male	188 (34.7)
Female	354 (65.3)
BMI ^1^	
Underweight	116 (21.4)
Normal	296 (54.6)
Overweight	66 (12.2)
Obese	64 (11.8)
Study Fields	
Health Sciences	203 (37.5)
Social and Humanities	182 (33.6)
Sciences and Technology	157 (29.0)
Academic Years	
Freshman	122 (22.5)
Sophomore	135 (24.9)
Junior	127 (23.4)
Senior	158 (29.2)
Living	
Parents	197 (36.3)
Roommates	210 (38.7)
Alone	135 (24.9)
Income (THB/month)	
≤5000	174 (32.1)
5001–10,000	271 (50.0)
>10,000	97 (17.9)
Online class	
≤3 days/week	261 (48.2)
>3 days/week	281 (51.8)
Digital usage	
<3 h/day	53 (9.8)
3–6 h/day	225 (41.5)
>6 h/day	264 (48.7)
Physical activity ^2^	
Sufficient	114 (21.0)
Insufficient	217 (40.0)
Inactivity	211 (38.9)
Smoking	
Smoking	15 (2.8)
No smoking	527 (97.2)
Cooking method	
By themself	29 (5.4)
Buying from outside	405 (74.7)
By others (parents, caregivers, or maids)	108 (19.9)
Perceived stress	
Low	317 (58.5)
Moderate	217 (40.0)
High	8 (1.5)
Quality of life	
Poor	13 (2.4)
Moderate	379 (69.9)
Good	150 (27.7)
Fruit intake	
<3 servings/day	536 (98.9)
≥3 servings/day	6 (1.1)
Vegetable intake	
<4 servings/day	540 (99.6)
≥4 servings/day	2 (0.4)

All *n* values are the number of observations. ^1^ BMI (body mass index); underweight defined as <18.5 kg/m^2^, normal as 18.5–22.9 kg/m^2^, overweight as 23.0–24.9 kg/m^2^, and obese as ≥25.0 kg/m^2^. ^2^ Physical activity ≥ 3 days/150 min per week is defined as sufficient, <3 days/150 min per week as insufficient, and no physical activity as inactivity.

**Table 2:** Binary regression of sociodemographic characteristics of fruit intake (*n* = 542).

Variables	Citrus Fruit		Pome Fruit		Berries and Other Small Fruits		Assorted Tropical and Subtropical Fruits (Edible Peel)		Assorted Tropical and Subtropical Fruits (Inedible Peel)		Melon Fruit	
	Adjusted OR (95% CI)		Adjusted OR (95% CI)		Adjusted OR (95% CI)		Adjusted OR (95% CI)		Adjusted OR (95% CI)		Adjusted OR (95% CI)	
	Yes	No	Yes	No	Yes	No	Yes	No	Yes	No	Yes	No
Sex (Reference = Male)												
Female	1.096 (0.664–1.810)	0.912 (0.553–1.506)	1.618 (1.086–2.410) *	0.618 (0.415–0.921) *	0.833 (0.544–1.276)	1.201 (0.784–1.839)	2.246 (1.499–3.363) ***	0.445 (0.297–0.667) ***	1.765 (0.961–3.239)	0.567 (0.309–1.040)	0.956 (0.647–1.413)	1.046 (0.708–1.546)
Living (Reference = Parents)												
Roommates	0.690 (0.38–1.253)	1.450 (0.798–2.635)	0.592 (0.37–0.946) *	1.689 (1.057–2.700) *	0.469 (0.283–0.776) **	2.133 (1.288–3.532) **	0.759 (0.467–1.233)	1.318 (0.811–2.140)	0.627 (0.289–1.359)	1.596 (0.736–3.463)	1.278 (0.805–2.027)	0.783 (0.493–1.242)
Alone	0.828 (0.43–1.597)	1.208 (0.626–2.328)	0.576 (0.342–0.969) *	1.736 (1.032–2.920) *	0.375 (0.210–0.670) ***	2.666 (1.493–4.760) ***	0.66 (0.387–1.126)	1.514 (0.888–2.581)	0.492 (0.218–1.114)	2.031 (0.898–4.594)	0.733 (0.439–1.223)	1.365 (0.817–2.279)
Cooking Method (Reference = Cooking by Themselves)												
Buying from outside	1.075 (0.379–3.051)	0.930 (0.328–2.641)	1.210 (0.545–2.689)	0.826 (0.372–1.836)	1.791 (0.673–4.765)	0.558 (0.210–1.486)	1.401 (0.624–3.147)	0.714 (0.318–1.603)	3.297 (1.291–8.425) *	0.303 (0.119–0.775) *	1.351 (0.609–2.998)	0.740 (0.334–1.643)
Cooking by parents, caregivers, or maids	2.728 (0.902–8.249)	0.367 (0.121–1.108)	1.566 (0.647–3.790)	0.638 (0.264–1.545)	2.818 (0.996–7.970)	0.355 (0.125–1.004)	1.407 (0.573–3.452)	0.711 (0.290–1.745)	7.176 (1.957–26.307) **	0.139 (0.038–0.511) **	1.887 (0.784–4.543)	0.53 (0.220–1.276)

* Sig. ≤ 0.05, ** Sig. ≤ 0.01, and *** Sig. ≤ 0.001.

**Table 3:** Binary regression of sociodemographic characteristics of vegetable intake (*n* = 542).

Variables	Cucurbitaceae		Brassicaceae		Solanaceae		Lamiaceae		Physalacriaceae		Fabaceae		Asteraceae	
	Adjusted OR (95% CI)		Adjusted OR (95% CI)		Adjusted OR (95% CI)		Adjusted OR (95% CI)		Adjusted OR (95% CI)		Adjusted OR (95% CI)		Adjusted OR (95% CI)	
	Yes	No	Yes	No	Yes	No	Yes	No	Yes	No	Yes	No	Yes	No
BMI (Reference = Obese)														
Underweight	0.592 (0.258–1.362)	1.690 (0.718–3.982)	1.100 (0.397–3.044)	0.909 (0.329–2.516)	0.672 (0.328–1.374)	1.489 (0.728–3.045)	0.467 (0.197–1.107)	2.139 (0.903–5.067)	0.254 (0.100–0.642) **	3.937 (1.557–9.953) **	0.573 (0.299–1.098)	1.744 (0.911–3.339)	0.611 (0.313–1.191)	1.637 (0.840–3.193)
Normal	0.788 (0.366–1.697)	1.199 (0.546–2.632)	1.785 (0.692–4.603)	0.560 (0.217–1.444)	0.677 (0.357–1.285)	1.477 (0.778–2.803)	0.333 (0.152–0.732) **	3.001 (1.366–6.593) **	0.352 (0.147–0.842) *	2.84 (1.188–6.790) *	0.734 (0.416–1.297)	1.362 (0.771–2.406)	0.83 (0.457–1.504)	1.205 (0.665–2.186)
Overweight	0.735 (0.286–1.886)	1.259 (0.471–3.367)	1.495 (0.384–5.827)	0.669 (0.172–2.606)	0.837 (0.358–1.960)	1.194 (0.510–2.796)	0.409 (0.155–1.075)	2.448 (0.930–6.441)	0.414 (0.142–1.203)	2.417 (0.831–7.027)	0.739 (0.354–1.542)	1.353 (0.649–2.821)	1.519 (0.670–3.444)	0.658 (0.290–1.493)
Academic Year (Reference = Senior)														
Freshman	1.334 (0.674–2.639)	0.808 (0.412–1.582)	2.408 (0.986–5.882)	0.415 (0.170–1.014)	1.053 (0.589–1.884)	0.950 (0.531–1.698)	0.750 (0.413–1.361)	1.334 (0.735–2.422)	1.088 (0.590–2.009)	0.919 (0.498–1.696)	1.216 (0.714–2.069)	0.822 (0.483–1.4)	0.780 (0.455–1.337)	1.282 (0.748–2.197)
Sophomore	1.425 (0.706–2.878)	0.756 (0.379–1.508)	2.334 (0.95–5.736)	0.428 (0.174–1.053)	0.85 (0.473–1.525)	1.177 (0.656–2.113)	0.904 (0.49–1.667)	1.106 (0.6–2.041)	1.327 (0.692–2.541)	0.754 (0.393–1.444)	1.344 (0.782–2.312)	0.744 (0.433–1.279)	1.058 (0.609–1.838)	0.945 (0.544–1.643)
Junior	0.945 (0.496–1.803)	1.074 (0.564–2.044)	3.660 (1.423–9.414) **	0.273 (0.106–0.703) **	0.869 (0.489–1.544)	1.151 (0.648–2.045)	0.924 (0.504–1.694)	1.082 (0.590–1.982)	1.119 (0.607–2.060)	0.894 (0.485–1.646)	1.585 (0.933–2.693)	0.631 (0.371–1.071)	0.953 (0.556–1.635)	1.049 (0.612–1.798)
Cooking Method (Reference = Cooking by Themself)														
Buying from outside	1.868 (0.758–4.606)	0.540 (0.220–1.324)	3.544 (1.242–10.113) *	0.282 (0.099–0.805) *	0.644 (0.255–1.632)	1.552 (0.613–3.929)	3.609 (1.578–8.253) **	0.277 (0.121–0.634) **	1.680 (0.696–4.060)	0.595 (0.246–1.438)	1.527 (0.667–3.497)	0.655 (0.286–1.499)	0.753 (0.319–1.778)	1.327 (0.562–3.134)
Cooking by parents, caregivers, or maids	2.571 (0.909–7.271)	0.402 (0.144–1.126)	3.675 (1.034–13.064) *	0.272 (0.077–0.967) *	0.824 (0.297–2.287)	1.213 (0.437–3.366)	3.195 (1.268–8.053) *	0.313 (0.124–0.789) *	2.261 (0.812–6.296)	0.442 (0.159–1.232)	2.120 (0.855–5.261)	0.472 (0.190–1.170)	0.913 (0.355–2.351)	1.095 (0.425–2.821)
Quality of Life (Reference = Poor)														
Mild	1.075 (0.268–4.312)	0.938 (0.235–3.752)	12.748 (3.201–50.779) ***	0.078 (0.02–0.312) ***	2.871 (0.878–9.389)	0.348 (0.107–1.139)	5.914 (1.645–21.266) **	0.169 (0.047–0.608) **	5.410 (1.548–18.905) *	0.185 (0.053–0.646) *	3.440 (0.857–13.812)	0.291 (0.072–1.168)	1.462 (0.435–4.914)	0.684 (0.203–2.299)
Good	1.303 (0.309–5.486)	0.774 (0.185–3.235)	9.783 (2.330–41.079) **	0.102 (0.024–0.429) **	2.76 (0.817–9.320)	0.362 (0.107–1.223)	7.068 (1.900–26.298) **	0.141 (0.038–0.526) **	4.372 (1.215–15.733) *	0.229 (0.064–0.823) *	3.462 (0.843–14.225)	0.289 (0.070–1.187)	1.421 (0.411–4.909)	0.704 (0.204–2.432)

* Sig. ≤ 0.05, ** Sig. ≤ 0.01, and *** Sig. ≤ 0.001.

**Table 4:** Reasons for fruit and vegetable consumption in undergraduate students.

Reason	Fruit Intake			Vegetable Intake		
	Total (*n* = 542)	No (*n* = 46)	Yes (*n* = 496)	Total (*n* = 540)	No (*n* = 9)	Yes (*n* = 531)
Taste (%)	85.4	60.9	87.7	54.8	33.3	55.2
Healthy (%)	77.9	56.5	79.8	80.7	55.6	81.2
Availability (%)	42.4	21.7	44.4	23.3	11.1	23.5
Other reason (%)	2.8	13.0	1.8	5.7	11.1	5.6

**Table 5:** Barriers to inadequate fruit and vegetable consumption (less than 400 g/d) in undergraduate students.

Barrier	Fruit Intake			Vegetable Intake		
	Total (*n* = 539)	No (*n* = 46)	Yes (*n* = 493)	Total (*n* = 541)	No (*n* = 9)	Yes (*n* = 532)
Sufficient intake perceived (%)	30.8	13.0	32.5	24.6	11.1	24.8
Unlike (%)	11.7	30.4	9.9	35.3	77.8	34.6
Preparation time needed (%)	22.3	13.0	23.1	17.4	22.2	17.3
Forgot to eat (%)	28.2	17.4	29.2	25.9	11.1	26.1
Expensive cost (%)	27.6	21.7	28.2	15.3	0.0	15.6
Having a busy lifestyle (%)	50.1	50.0	50.1	56.9	11.1	57.7
Unknown benefits of FVs (%)	1.5	2.2	1.4	1.8	0.0	1.9
Other barriers (%)	0.7	0.0	0.8	0.9	0.0	0.9

## Data Availability

Data is contained within the article and Supplementary Materials.

## Supplementary Materials

The following supporting information can be downloaded at: https://www.mdpi.com/article/10.3390/nu16060779/s1, Figure S1: example of fruit portion size provided in the questionnaire for estimating the amount of each fruit variety consumption; Figure S2: example of vegetable portion size provided in the questionnaire for estimating the amount of each vegetable variety consumption; Table S1: list of each fruit and vegetable variety; Table S2: fruit form purchased by undergraduate students; Table S3: vegetable form purchased by undergraduate students; Table S4: association of sociodemographic characteristics of fruit intake; Table S5: association of sociodemographic characteristics of vegetable intake; Table S6: binary logistic regression of sociodemographic characteristics associated with each type of fruit intake among undergraduate students; Table S7: binary logistic regression of sociodemographic characteristics associated with each type of vegetable intake among undergraduate students.

## References

[1] Liu R.H. (2013). Health-promoting components of fruits and vegetables in the diet. Adv. Nutr..

[2] Chen L., Pu Y., Xu Y., He X., Cao J., Ma Y., Jiang W. (2022). Anti-diabetic and anti-obesity: Efficacy evaluation and exploitation of polyphenols in fruits and vegetables. Food Res. Int..

[3] Hung H.C., Joshipura K.J., Jiang R., Hu F.B., Hunter D., Smith-Warner S.A., Colditz G.A., Rosner B., Spiegelman D., Willett W.C. (2004). Fruit and vegetable intake and risk of major chronic disease. J. Natl. Cancer Inst..

[4] Larsson S.C., Virtamo J., Wolk A. (2013). Total and specific fruit and vegetable consumption and risk of stroke: A prospective study. Atherosclerosis.

[5] Who J., Consultation F.E. (2003). Diet, Nutrition and the Prevention of Chronic Diseases.

[6] Wallace T.C., Bailey R.L., Blumberg J.B., Burton-Freeman B., Chen C.O., Crowe-White K.M., Drewnowski A., Hooshmand S., Johnson E., Lewis R. (2020). Fruits, vegetables, and health: A comprehensive narrative, umbrella review of the science and recommendations for enhanced public policy to improve intake. Crit. Rev. Food Sci. Nutr..

[7] Aune D., Giovannucci E., Boffetta P., Fadnes L.T., Keum N., Norat T., Greenwood D.C., Riboli E., Vatten L.J., Tonstad S. (2017). Fruit and vegetable intake and the risk of cardiovascular disease, total cancer and all-cause mortality-a systematic review and dose-response meta-analysis of prospective studies. Int. J. Epidemiol..

[8] Smith L., López Sánchez G.F., Veronese N., Soysal P., Oh H., Barnett Y., Keyes H., Butler L., Allen P., Kostev K. (2022). Fruit and Vegetable Intake and Non-Communicable Diseases among Adults Aged ≥ 50 Years in Low- and Middle-Income Countries. J. Nutr. Health Aging.

[9] Krebs-Smith S.M., Guenther P.M., Subar A.F., Kirkpatrick S.I., Dodd K.W. (2010). Americans do not meet federal dietary recommendations. J. Nutr..

[10] Baghurst K. (1999). Red meat consumption in Australia: Intakes, contributions to nutrient intake and associated dietary patterns. Eur. J. Cancer Prev..

[11] Kucuk N., Urak F., Bilgic A., Florkowski W.J., Kiani A.K., Ozdemir F.N. (2023). Fruit and vegetable consumption across population segments: Evidence from a national household survey. J. Health Popul. Nutr..

[12] Poobalan A.S., Aucott L.S., Clarke A., Smith W.C. (2014). Diet behaviour among young people in transition to adulthood (18–25 year olds): A mixed method study. Health Psychol. Behav. Med..

[13] Albani V., Butler L.T., Traill W.B., Kennedy O.B. (2017). Fruit and vegetable intake: Change with age across childhood and adolescence. Br. J. Nutr..

[14] Bull F.C., Al-Ansari S.S., Biddle S., Borodulin K., Buman M.P., Cardon G., Carty C., Chaput J.P., Chastin S., Chou R. (2020). World Health Organization 2020 guidelines on physical activity and sedentary behaviour. Br. J. Sports Med..

[15] (2016). Classification of Agricultural Commodities: Crop.

[16] World Health Organization (2018). Increasing Fruit and Vegetable Consumption to Reduce the Risk of Non-Communicable Diseases.

[17] Wongpakaran N., Wongpakaran T. (2010). The Thai version of the PSS-10: An Investigation of its psychometric properties. Biopsychosoc. Med..

[18] Mahatnirundkul S. (1998). Comparison of the WHOQOL-100 and the WHOQOL-BREF (26 items). J. Ment. Health Thai.

[19] Toh D.W.K., Koh E.S., Kim J.E. (2020). Incorporating healthy dietary changes in addition to an increase in fruit and vegetable intake further improves the status of cardiovascular disease risk factors: A systematic review, meta-regression, and meta-analysis of randomized controlled trials. Nutr. Rev..

[20] Duthie S.J., Duthie G.G., Russell W.R., Kyle J.A.M., Macdiarmid J.I., Rungapamestry V., Stephen S., Megias-Baeza C., Kaniewska J.J., Shaw L. (2018). Effect of increasing fruit and vegetable intake by dietary intervention on nutritional biomarkers and attitudes to dietary change: A randomised trial. Eur. J. Nutr..

[21] Hilger J., Loerbroks A., Diehl K. (2017). Eating behaviour of university students in Germany: Dietary intake, barriers to healthy eating and changes in eating behaviour since the time of matriculation. Appetite.

[22] Vilaro M.J., Colby S.E., Riggsbee K., Zhou W., Byrd-Bredbenner C., Olfert M.D., Barnett T.E., Horacek T., Sowers M., Mathews A.E. (2018). Food Choice Priorities Change Over Time and Predict Dietary Intake at the End of the First Year of College Among Students in the U.S. Nutrients.

[23] Wellard-Cole L., Watson W.L., Hughes C., Tan N., Dibbs J., Edge R., Dessaix A. (2023). Perceptions of adequacy of fruit and vegetable intake as a barrier to increasing consumption. Nutr. Diet.

[24] Alperet D.J., Butler L.M., Koh W.-P., Yuan J.-M., van Dam R.M. (2017). Influence of temperate, subtropical, and tropical fruit consumption on risk of type 2 diabetes in an Asian population1, 2, 3. Am. J. Clin. Nutr..

[25] Schösler H., de Boer J., Boersema J.J., Aiking H. (2015). Meat and masculinity among young Chinese, Turkish and Dutch adults in the Netherlands. Appetite.

[26] Papadaki A., Hondros G., Scott J.A., Kapsokefalou M. (2007). Eating habits of university students living at, or away from home in Greece. Appetite.

[27] El Ansari W., Stock C., Mikolajczyk R.T. (2012). Relationships between food consumption and living arrangements among university students in four European countries—A cross-sectional study. Nutr. J..

[28] Bere E., Klepp K.I. (2005). Changes in accessibility and preferences predict children’s future fruit and vegetable intake. Int. J. Behav. Nutr. Phys. Act..

[29] Kremmyda L.-S., Papadaki A., Hondros G., Kapsokefalou M., Scott J.A. (2008). Differentiating between the effect of rapid dietary acculturation and the effect of living away from home for the first time, on the diets of Greek students studying in Glasgow. Appetite.

[30] Mills S., White M., Brown H., Wrieden W., Kwasnicka D., Halligan J., Robalino S., Adams J. (2017). Health and social determinants and outcomes of home cooking: A systematic review of observational studies. Appetite.

[31] Ashworth A., Mitchell K., Blackwell J.R., Vanhatalo A., Jones A.M. (2015). High-nitrate vegetable diet increases plasma nitrate and nitrite concentrations and reduces blood pressure in healthy women. Public Health Nutr..

[32] Sobko T., Marcus C., Govoni M., Kamiya S. (2010). Dietary nitrate in Japanese traditional foods lowers diastolic blood pressure in healthy volunteers. Nitric Oxide.

[33] Murphy M.M., Barraj L.M., Spungen J.H., Herman D.R., Randolph R.K. (2014). Global assessment of select phytonutrient intakes by level of fruit and vegetable consumption. Br. J. Nutr..

[34] Nagai A., Kubota M., Morinaga K., Higashiyama Y. (2017). Food acceptance and anthropometry in relation to 6-n-propylthiouracil sensitivity in Japanese college women. Asia Pac. J. Clin. Nutr..

[35] Al-Awwad N.J., Al-Sayyed H.F., Zeinah Z.A., Tayyem R.F. (2021). Dietary and lifestyle habits among university students at different academic years. Clin. Nutr. ESPEN.

[36] Deshpande S., Basil M.D., Basil D.Z. (2009). Factors Influencing Healthy Eating Habits Among College Students: An Application of the Health Belief Model. Health Mark. Q..

[37] Matar Boumosleh J., Jaalouk D. (2017). Depression, anxiety, and smartphone addiction in university students—A cross sectional study. PLoS ONE.

[38] Choi J. (2020). Impact of Stress Levels on Eating Behaviors among College Students. Nutrients.

[39] Deliens T., Verhoeven H., De Bourdeaudhuij I., Huybrechts I., Mullie P., Clarys P., Deforche B. (2018). Factors associated with fruit and vegetable and total fat intake in university students: A cross-sectional explanatory study. Nutr. Diet..

[40] Whatnall M.C., Soo Z.M., Patterson A.J., Hutchesson M.J. (2021). University Students Purchasing Food on Campus More Frequently Consume More Energy-Dense, Nutrient-Poor Foods: A Cross-Sectional Survey. Nutrients.

[41] Wagner M.G., Rhee Y., Honrath K., Blodgett Salafia E.H., Terbizan D. (2016). Nutrition education effective in increasing fruit and vegetable consumption among overweight and obese adults. Appetite.

[42] Ribeiro-Silva R.C., Fiaccone R.L., Conceicao-Machado M., Ruiz A.S., Barreto M.L., Santana M.L.P. (2018). Body image dissatisfaction and dietary patterns according to nutritional status in adolescents. J. Pediatr..

[43] Menezes M.C., Diez Roux A.V., Souza Lopes A.C. (2018). Fruit and vegetable intake: Influence of perceived food environment and self-efficacy. Appetite.

[44] Gibbons C. (2022). Understanding the role of stress, personality and coping on learning motivation and mental health in university students during a pandemic. BMC Psychol..

[45] Duchscher J.E. (2009). Transition shock: The initial stage of role adaptation for newly graduated registered nurses. J. Adv. Nurs..

[46] Spivey C.A., Stallworth S., Olivier E., Chisholm-Burns M.A. (2020). Examination of the Relationship between Health-related Quality of Life and Academic Performance Among Student Pharmacists. Curr. Pharm. Teach. Learn..

[47] Enns S.C., Perotta B., Paro H.B., Gannam S., Peleias M., Mayer F.B., Santos I.S., Menezes M., Senger M.H., Barelli C. (2016). Medical Students’ Perception of Their Educational Environment and Quality of Life: Is There a Positive Association?. Acad. Med..

[48] Costa D.G., Carleto C.T., Santos V.S., Haas V.J., Goncalves R., Pedrosa L.A.K. (2018). Quality of life and eating attitudes of health care students. Rev. Bras. Enferm..

[49] Chusak C., Tangmongkhonsuk M., Sudjapokinon J., Adisakwattana S. (2022). The Association between Online Learning and Food Consumption and Lifestyle Behaviors and Quality of Life in Terms of Mental Health of Undergraduate Students during COVID-19 Restrictions. Nutrients.

[50] Chachula K.M., Ahmad N. (2022). Professional quality of life, stress, and trauma in nursing students: Before and during the novel coronavirus pandemic. Psychol. Trauma.

[51] Micha R., Karageorgou D., Bakogianni I., Trichia E., Whitsel L.P., Story M., Penalvo J.L., Mozaffarian D. (2018). Effectiveness of school food environment policies on children’s dietary behaviors: A systematic review and meta-analysis. PLoS ONE.

[52] Majid H.A., Ng A.K., Dahlui M., Mohammadi S., Mohamed M., Su T.T., Jalaludin M.Y. (2022). Outcome Evaluation on Impact of the Nutrition Intervention among Adolescents: A Feasibility, Randomised Control Study from Myheart Beat (Malaysian Health and Adolescents Longitudinal Research Team-Behavioural Epidemiology and Trial). Nutrients.

[53] Zhang J., Oh Y.J., Lange P., Yu Z., Fukuoka Y. (2020). Artificial Intelligence Chatbot Behavior Change Model for Designing Artificial Intelligence Chatbots to Promote Physical Activity and a Healthy Diet: Viewpoint. J. Med. Internet Res..

[54] Deliens T., Van Crombruggen R., Verbruggen S., De Bourdeaudhuij I., Deforche B., Clarys P. (2016). Dietary interventions among university students: A systematic review. Appetite.

