# Supplementary material for: Examining Sociodemographic Factors, Reasons, and Barriers in the Diversity of Fruit and Vegetable Intake among Undergraduate Students
Source: Nutrients. 2024 Mar 9;16(6):779. doi: 10.3390/nu16060779 (PMC10976196; doi:10.3390/nu16060779)

Supplementary Figure S1 The example of fruit portion size provided in the questionnaire for estimating the amount of each fruit variety consumption.

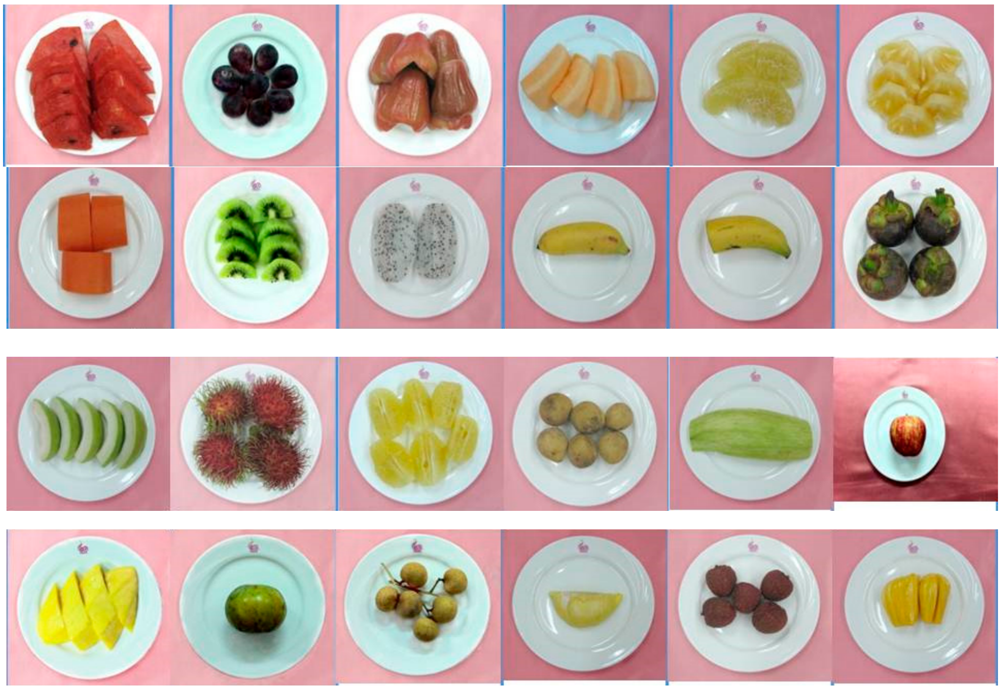

Supplementary Figure S2 The example of vegetable portion size provided in the questionnaire for estimating the amount of each vegetable variety consumption.

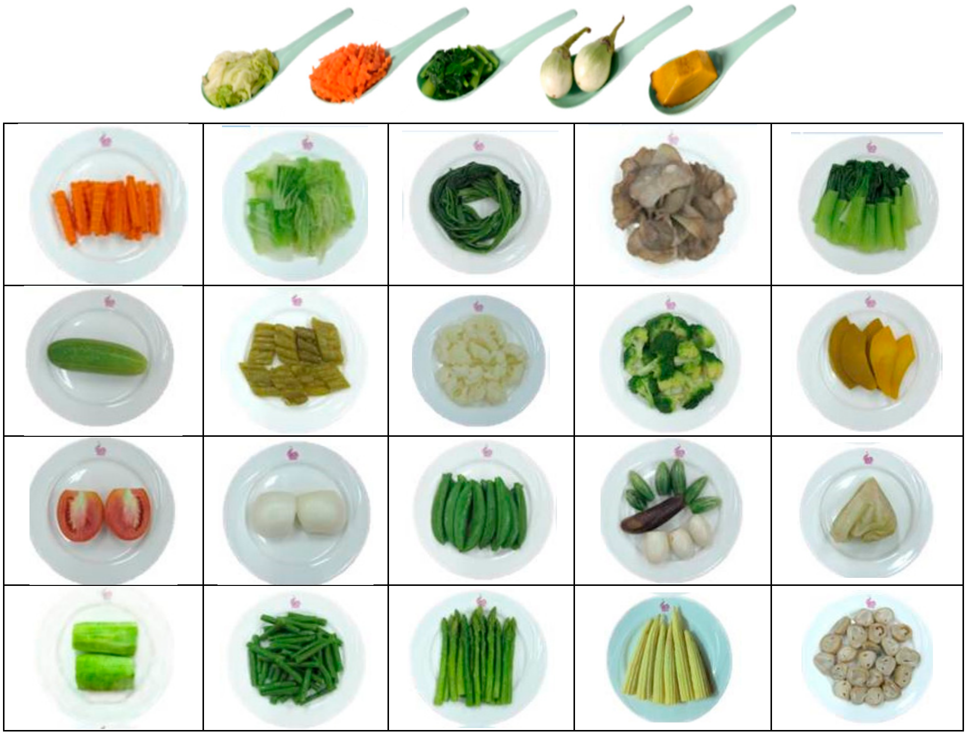

Supplement: Supplementary file 1 [file nutrients-16-00779-s001.zip › Supplementary Figure S1-S2.pdf]
