# Supplementary material for: Examining Sociodemographic Factors, Reasons, and Barriers in the Diversity of Fruit and Vegetable Intake among Undergraduate Students
Source: Nutrients. 2024 Mar 9;16(6):779. doi: 10.3390/nu16060779 (PMC10976196; doi:10.3390/nu16060779)
Supplement: Supplementary file 1 [file nutrients-16-00779-s001.zip › Supplementary Table S1-S7.pdf]

Supplementary Table S1 The list of each fruit and vegetable variety.

Varieties	Example
**Fruits**	
1. Citrus fruit	Pomelo
2. Pome fruit	Apple
3. Berries and other small fruit	Grape
4. Assorted tropical and subtropical fruits (edible peel)	Santol, rose apple, jujube, guava
5. Assorted tropical and subtropical fruits (inedible peel)	Longan, durian, mangosteen, lichee, mango, rambutan, pineapple, coconut, dragon fruit, custard apple, longkang, jackfruit, zalaca, banana
6. Melon fruit	Cantaloup, watermelon
**Vegetables**	
1. *Cucurbitaceae*	Cucumber, gourd, pumpkin, squash, bitter gourd, grandis
2. *Brassicaceae*	Cabbage, cauliflower, kale, broccoli, choi, chinese cabbage, radish
3. *Solanaceae*	Tomato, potato
4. *Lamiaceae*	Basil
5. *Physalacriaceae*	Mushroom
6. *Fabaceae*	Cowpea
7. *Asteraceae*	Lettuce

Supplementary Table S2 Fruit form purchased by undergraduate students.

**Purchased form**	**Fruit intake**		
	Total (n = 538)	No (n = 43)	Yes (n = 495)
Raw fruit (%)	100.0	100.0	100.0
Processed fruit (%)	39.0	32.6	39.6

Supplementary Table S3 Vegetable form purchased by undergraduate students.

**Purchased form**	**vegetable intake**		
	Total (n = 541)	No (n = 9)	Yes (n = 532)
Cooked vegetable (%)	95.2	88.9	95.3
Raw vegetable (%)	65.6	33.3	66.2
Processed vegetable (%)	26.6	N/A	27.1

Supplementary Table S4 The association of sociodemographic characteristics of fruit intake (*n* = 542).

Variables	Citrus fruit			Pone fruit			Berries and other small fruit			Assorted tropical and subtropical fruits (edible peel)			Assorted tropical and subtropical fruits (inedible peel)			Melon fruit		
	No (%)	Yes (%)	*p*-value	No (%)	Yes (%)	*p*-value	No (%)	Yes (%)	*p*-value	No (%)	Yes (%)	*p*-value	No (%)	Yes (%)	*p*-value	No (%)	Yes (%)	*p*-value
**Sex**																		
Male	80.9	19.1	0.801	56.4	43.6	0.030*	65.4	34.6	0.167	52.1	47.9	<0.001***	15.4	84.6	0.057	47.9	52.1	0.510
Female	79.9	20.1		46.6	53.4		71.2	28.8		33.6	66.4		9.9	90.1		33.3	36.0	
**BMI**																		
Underweight	81.9	18.1	0.179	55.2	44.8	0.268	71.6	28.4	0.406	37.9	62.1	0.809	12.1	87.9	0.521	51.7	48.3	0.467
Normal	77.7	22.3		46.3	53.7		66.6	33.4		41.6	58.4		12.5	87.5		48.3	51.7	
Overweight	89.4	10.6		51.5	48.5		69.7	30.3		40.9	59.1		13.6	86.4		57.6	42.4	
Obese	79.7	20.3		56.3	43.8		76.6	23.4		35.9	64.1		6.3	93.8		45.3	54.7	
**Study Fields**																		
Health Sciences	79.3	20.7	0.517	45.8	54.2	0.274	72.4	27.6	0.091	35.0	65.0	0.175	10.8	89.2	0.849	50.7	49.3	0.481
Social and Humanities	83.0	17.0		51.1	48.9		71.4	28.6		43.4	56.6		12.1	87.9		52.2	47.8	
Sciences and Technology	78.3	21.7		54.1	45.9		62.4	37.6		42.7	57.3		12.7	87.3		45.9	54.1	
**Academic Years**																		
Freshman	76.2	23.8	0.503	46.7	53.3	0.438	63.9	36.1	0.182	40.2	59.8	0.893	13.9	86.1	0.363	46.7	53.3	0.659
Sophomore	79.3	20.7		53.3	46.7		67.4	32.6		37.8	62.2		14.8	85.2		51.1	48.9	
Junior	83.5	16.5		45.7	54.3		76.4	23.6		42.5	57.5		9.4	90.6		47.2	52.8	
Senior	81.6	18.4		53.2	46.8		69.0	31.0		39.9	60.1		9.5	90.5		53.2	46.8	
**Living**																		
Parents	74.1	25.9	0.021*	39.6	60.4	0.001***	58.4	41.6	<0.001***	34.0	66.0	0.084	7.6	92.4	0.046*	48.7	51.3	0.134
Roommates	84.8	15.2		55.2	44.8		73.8	26.2		42.4	57.6		12.9	87.1		46.2	53.8	
Alone	82.2	17.8		57.0	43.0		77.8	22.2		45.2	54.8		16.3	83.7		57.0	43.0	
**Income**																		
≤ 5,000 bath/month	79.9	20.1	0.325	50.0	50.0	0.939	73.0	27.0	0.395	35.6	64.4	0.179	12.1	87.9	0.470	52.3	47.7	0.638
5,001-10,000 bath/month	82.3	17.7		49.4	50.6		67.9	32.1		43.9	56.1		12.9	87.1		49.4	50.6	
>10,000 bath/month	75.3	24.7		51.5	48.5		66.0	34.0		37.1	62.9		8.2	91.8		46.4	53.6	
**Online class**																		
≤ 3 days/week	83.1	16.9	0.104	47.1	52.9	0.197	69.7	30.3	0.792	42.5	57.5	0.254	10.3	89.7	0.309	51.0	49.0	0.608
> 3 days/week	77.6	22.4		52.7	47.3		68.7	31.3		37.7	62.3		13.2	86.8		48.8	51.2	
**Digital usage**																		

< 3 hours/day	77.4	22.6		35.8	64.2		62.3	37.7		32.1	67.9		3.8	96.2		56.6	43.4	
3-6 hours/day	80.4	19.6	0.854	52.0	48.0	0.093	67.6	32.4	0.297	42.7	57.3	0.351	13.3	86.7	0.149	52.4	47.6	0.226
> 6 hours/day	80.7	19.3		51.1	48.9		72.0	28.0		39.4	60.6		12.1	87.9		46.2	53.8	
**Physical activity**																		
Sufficient	77.2	22.8		45.6	54.4		65.8	34.2		41.2	58.8		13.2	86.8		44.7	55.3	
Insufficient	80.6	19.4	0.635	46.1	53.9	0.059	68.7	31.3	0.548	35.9	64.1	0.259	10.1	89.9	0.613	49.3	50.7	0.350
Inactivity	81.5	18.5		56.4	43.6		71.6	28.4		43.6	56.4		12.8	87.2		53.1	46.9	
**Smoking**																		
Smoking	80.0	20.0	0.980	53.3	46.7	0.793	73.3	26.7	0.724	60.0	40.0	0.110	0.0	100.0	0.151	53.3	46.7	0.782
No smoking	80.3	19.7		49.9	50.1		69.1	30.9		39.5	60.5		12.1	87.9		49.7	50.3	
**Cooking method**																		
by themself	82.8	17.2		55.2	44.8		79.3	20.7		48.3	51.7		31.0	69.0		58.6	41.4	
Buying from outside	83.7	16.3	<0.001***	52.3	47.7	0.058	71.9	28.1	0.004**	41.0	59.0	0.290	12.3	87.7	<0.001***	50.6	49.4	0.325
by others (parents, caregivers, or maids)	66.7	33.3		39.8	60.2		56.5	43.5		34.3	65.7		4.6	95.4		44.4	55.6	
**Stress**																		
Low	79.5	20.5		51.7	48.3		68.1	31.9		42.6	57.4		12.6	87.4		52.4	47.6	
Moderate	80.6	19.4	0.349	47.5	52.5	0.625	70.5	29.5	0.792	35.5	64.5	0.110	10.1	89.9	0.347	47.0	53.0	0.175
High	100.0	0.0		50.0	50.0		75.0	25.0		62.5	37.5		25.0	75.0		25.0	75.0	
**Quality of life**																		
Poor	76.9	23.1		76.9	23.1		76.9	23.1		53.8	46.2		30.8	69.2		53.8	46.2	
Mild	80.7	19.3	0.892	50.7	49.3	0.091	69.7	30.3	0.724	40.4	59.6	0.520	11.6	88.4	0.096	49.3	50.7	0.922
Good	79.3	20.7		46.0	54.0		67.3	32.7		38.0	62.0		10.7	89.3		50.7	49.3	

All values are represented as percentages of undergraduate students, and *P*-values were determined using the chi-square test.

Supplementary Table S5 The association of sociodemographic characteristics of vegetable intake (n = 542).

Variables	Cucurbitaceae			Brassicaceae			Solanaceae			Lamiaceae			Physalacriaceae			Fabaceae			Asteraceae		
	No (%)	Yes (%)	*p*-value	No (%)	Yes (%)	*p*-value	No (%)	Yes (%)	*p*-value	No (%)	Yes (%)	*p*-value	No (%)	Yes (%)	*p*-value	No (%)	Yes (%)	*p*-value	No (%)	Yes (%)	*p*-value
**Sex**																					
Male	18.1	81.9	0.934	8.5	91.5	0.754	24.5	75.5	0.070	19.1	80.9	0.004**	24.5	75.5	0.426	52.1	47.9	0.156	42.0	58.0	0.062
Female	17.8	82.2		9.3	90.7		31.9	68.1		30.5	69.5		21.5	78.5		58.5	41.5		33.9	66.1	
**BMI**																					
Underweight	21.6	78.4	0.618	12.1	87.9	0.318	32.8	67.2	0.501	25.0	75.0	0.050*	29.3	70.7	0.032*	62.1	37.9	0.353	44.8	55.2	0.029*
Normal	17.2	82.8		7.8	92.2		30.1	69.9		30.4	69.6		23.3	76.7		56.1	43.9		37.2	62.8	
Overweight	18.2	81.8		6.1	93.9		22.7	77.3		24.2	75.8		18.2	81.8		54.5	45.5		22.7	77.3	
Obese	14.1	85.9		12.5	87.5		26.6	73.4		14.1	85.9		10.9	89.1		48.4	51.6		34.4	65.6	
**Study Fields**																					
Health Sciences	17.2	82.8	0.708	7.4	92.6	0.469	31.0	69.0	0.162	25.6	74.4	0.246	17.2	82.8	0.012*	57.1	42.9	0.704	35.5	64.5	0.188
Social and Humanities	19.8	80.2		11.0	89.0		32.4	67.6		30.8	69.2		29.7	70.3		57.7	42.3		41.8	58.2	
Sciences and Technology	16.6	83.4		8.9	91.1		23.6	76.4		22.9	77.1		21.0	79.0		53.5	46.5		32.5	67.5	
**Academic Years**																					
Freshman	19.7	80.3	0.606	15.6	84.4	0.030*	27.9	72.1	0.874	26.2	73.8	0.912	27.0	73.0	0.411	62.3	37.7	0.468	37.7	62.3	0.745
Sophomore	16.3	83.7		7.4	92.6		27.4	72.6		28.9	71.1		23.0	77.0		54.8	45.2		40.0	60.0	
Junior	15.0	85.0		8.7	91.3		30.7	69.3		26.0	74.0		18.1	81.9		55.9	44.1		33.9	66.1	
Senior	20.3	79.7		5.7	94.3		31.0	69.0		25.3	74.7		22.2	77.8		53.2	46.8		35.4	64.6	
**Living**																					
Parents	19.3	80.7	0.574	8.1	91.9	0.853	26.4	73.6	0.085	27.9	72.1	0.675	17.8	82.2	0.132	60.4	39.6	0.329	35.5	64.5	0.415
Roommates	15.7	84.3		9.5	90.5		34.8	65.2		27.1	72.9		24.8	75.2		53.3	46.7		40.0	60.0	
Alone	19.3	80.7		9.6	90.4		25.2	74.8		23.7	76.3		25.9	74.1		54.8	45.2		33.3	66.7	
**Income**																					
≤ 5,000 bath/month	14.4	85.6	0.338	9.8	90.2	0.526	35.1	64.9	0.129	27.6	72.4	0.879	23.0	77.0	0.582	48.9	51.1	0.051	38.5	61.5	0.047*
5,001-10,000 bath/month	19.6	80.4		7.7	92.3		26.9	73.1		26.6	73.4		23.6	76.4		60.5	39.5		39.5	60.5	
>10,000 bath/month	19.6	80.4		11.3	88.7		25.8	74.2		24.7	75.3		18.6	81.4		57.7	42.3		25.8	74.2	
**Online class**																					
≤ 3 days/week	18.8	81.2	0.608	8.8	91.2	0.858	31.0	69.0	0.403	28.0	72.0	0.477	25.3	74.7	0.136	61.7	38.3	0.014*	40.2	59.8	0.102

> 3 days/week	17.1	82.9		9.3	90.7		27.8	72.2		25.3	74.7		19.9	80.1		51.2	48.8		33.5	66.5	
**Digital usage**																					
< 3 hours/day	15.1	84.9		3.8	96.2		15.1	84.9		24.5	75.5		18.9	81.1		54.7	45.3		35.8	64.2	
3-6 hours/day	17.8	82.2	0.833	9.3	90.7	0.364	31.1	68.9	0.056	27.1	72.9	0.929	21.3	78.7	0.596	59.1	40.9	0.531	36.4	63.6	0.979
> 6 hours/day	18.6	81.4		9.8	90.2		30.7	69.3		26.5	73.5		24.2	75.8		54.2	45.8		37.1	62.9	
**Physical activity**																					
Sufficient	20.2	79.8		9.6	90.4		23.7	76.3		28.9	71.1		25.4	74.6		57.0	43.0		36.8	63.2	
Insufficient	15.2	84.8	0.405	6.5	93.5	0.200	26.3	73.7	0.036*	21.7	78.3	0.103	18.4	81.6	0.178	50.7	49.3	0.074	29.5	70.5	0.007**
Inactivity	19.4	80.6		11.4	88.6		35.5	64.5		30.3	69.7		25.1	74.9		61.6	38.4		44.1	55.9	
**Smoking**																					
Smoking	13.3	86.7	0.640	0.0	100.0	0.216	13.3	86.7	0.167	13.3	86.7	0.239	13.3	86.7	0.388	46.7	53.3	0.447	26.7	73.3	0.413
No smoking	18.0	82.0		9.3	90.7		29.8	70.2		26.9	73.1		22.8	77.2		56.5	43.5		37.0	63.0	
**Cooking method**																					
By themself	27.6	72.4		24.1	75.9		24.1	75.9		51.7	48.3		34.5	65.5		65.5	34.5		31.0	69.0	
Buying from outside	17.8	82.2	0.333	8.4	91.6	0.014*	30.4	69.6	0.635	24.4	75.6	0.005**	23.2	76.8	0.100	56.3	43.7	0.523	37.8	62.2	0.644
By others (parents, caregivers, or maids)	15.7	84.3		7.4	92.6		26.9	73.1		27.8	72.2		16.7	83.3		53.7	46.3		34.3	65.7	
**Stress**																					
Low	18.3	81.7		9.8	90.2		29.3	70.7		26.2	73.8		23.3	76.7		54.6	45.4		36.0	64.0	
Moderate	17.1	82.9	0.813	7.8	92.2	0.701	29.0	71.0	0.875	27.2	72.8	0.962	21.2	78.8	0.832	58.5	41.5	0.623	38.2	61.8	0.681
High	25.0	75.0		12.5	87.5		37.5	62.5		25.0	75.0		25.0	75.0		62.5	37.5		25.0	75.0	
**Quality of life**																					
Poor	23.1	76.9		46.2	53.8		46.2	53.8		61.5	38.5		53.8	46.2		76.9	23.1		38.5	61.5	
Mild	18.2	81.8	0.812	7.4	92.6	<0.001***	29.0	71.0	0.402	26.4	73.6	0.013*	20.8	79.2	0.017*	55.4	44.6	0.305	36.9	63.1	0.971
Good	16.7	83.3		10.0	90.0		28.7	71.3		24.0	76.0		24.0	76.0		56.7	43.3		36.0	64.0	

All values are represented as percentages of undergraduate students, and *P*-values were determined using the chi-square test.

Supplementary Table S6 Binary logistic regression of socio-demographic characteristics associated with each type of fruit intake among undergraduate students (*n* = 542).

Variables	Citrus fruit		Pone fruit		Berries and other small fruits		Assorted tropical and subtropical fruits (edible peel)		Assorted tropical and subtropical fruits (inedible peel)		Melon fruit	
	Adjusted OR (95% CI)		Adjusted OR (95% CI)		Adjusted OR (95% CI)		Adjusted OR (95% CI)		Adjusted OR (95% CI)		Adjusted OR (95% CI)	
	Yes	No	Yes	No	Yes	No	Yes	No	Yes	No	Yes	No
**Sex (Reference = Male)**												
**Female**	1.096 (0.664-1.810)	0.912 (0.553-1.506)	1.618 (1.086-2.410)*	0.618 (0.415-0.921)*	0.833 (0.544-1.276)	1.201 (0.784-1.839)	2.246 (1.499-3.363)***	0.445 (0.297-0.667)***	1.765 (0.961-3.239)	0.567 (0.309-1.040)	0.956 (0.647-1.413)	1.046 (0.708-1.546)
**Living (Reference = parents)**												
**Roommates**	0.690 (0.380-1.253)	1.450 (0.798-2.635)	0.592 (0.370-0.946)*	1.689 (1.057-2.700)*	0.469 (0.283-0.776)**	2.133 (1.288-3.532)**	0.759 (0.467-1.233)	1.318 (0.811-2.14)	0.627 (0.289-1.359)	1.596 (0.736-3.463)	1.278 (0.805-2.027)	0.783 (0.493-1.242)
**Alone**	0.828 (0.430-1.597)	1.208 (0.626-2.328)	0.576 (0.342-0.969)*	1.736 (1.032-2.920)*	0.375 (0.210-0.670)***	2.666 (1.493-4.760)***	0.66 (0.387-1.126)	1.514 (0.888-2.581)	0.492 (0.218-1.114)	2.031 (0.898-4.594)	0.733 (0.439-1.223)	1.365 (0.817-2.279)
**Cooking method (Reference = by themself)**												
**Buying from outside**	1.075 (0.379-3.051)	0.930 (0.328-2.641)	1.210 (0.545-2.689)	0.826 (0.372-1.836)	1.791 (0.673-4.765)	0.558 (0.210-1.486)	1.401 (0.624-3.147)	0.714 (0.318-1.603)	3.297 (1.291-8.425)*	0.303 (0.119-0.775)*	1.351 (0.609-2.998)	0.740 (0.334-1.643)
**By parents**	2.728 (0.902-8.249)	0.367 (0.121-1.108)	1.566 (0.647-3.790)	0.638 (0.264-1.545)	2.818 (0.996-7.970)*	0.355 (0.125-1.004)*	1.407 (0.573-3.452)	0.711 (0.290-1.745)	7.176 (1.957-26.307)**	0.139 (0.038-0.511)**	1.887 (0.784-4.543)	0.53 (0.220-1.276)

* Sig. ≤ 0.05, ** Sig. ≤ 0.01, *** Sig. ≤ 0.001

Supplementary Table S7 Binary logistic regression of socio-demographic characteristics associated with each type of vegetable intake among undergraduate students (*n* = 542).

**Variables**	**Cucurbitaceae**		**Brassicaceae**		**Solanaceae**		**Lamiaceae**		**Physalacriaceae**		**Fabaceae**		**Asteraceae**	
	Adjusted OR (95% CI)		Adjusted OR (95% CI)		Adjusted OR (95% CI)		Adjusted OR (95% CI)		Adjusted OR (95% CI)		Adjusted OR (95% CI)		Adjusted OR (95% CI)	
	Yes	No	Yes	No	Yes	No	Yes	No	Yes	No	Yes	No	Yes	No
**Sex (Reference = Male)**														
**Female**	1.063 (0.636-1.775)	0.981 (0.589-1.634)	1.050 (0.511-2.157)	0.952 (0.464-1.956)	0.901 (0.579-1.401)	1.110 (0.714-1.727)	0.528 (0.326-0.858)**	1.892 (1.166-3.071)**	1.273 (0.789-2.055)	0.785 (0.487-1.267)	0.795 (0.535-1.183)	1.257 (0.845-1.87)	1.786 (1.184-2.693)**	0.560 (0.371-0.844)**
**BMI classification 2 (Reference = Obese)**														
**Underweight**	0.592 (0.258-1.362)	1.690 (0.718-3.982)	1.100 (0.397-3.044)	0.909 (0.329-2.516)	0.672 (0.328-1.374)	1.489 (0.728-3.045)	0.467 (0.197-1.107)	2.139 (0.903-5.067)	0.254 (0.100-0.642)**	3.937 (1.557-9.953)**	0.573 (0.299-1.098)	1.744 (0.911-3.339)	0.611 (0.313-1.191)	1.637 (0.840-3.193)
**Normal**	0.788 (0.366-1.697)	1.199 (0.546-2.632)	1.785 (0.692-4.603)	0.560 (0.217-1.444)	0.677 (0.357-1.285)	1.477 (0.778-2.803)	0.333 (0.152-0.732)**	3.001 (1.366-6.593)**	0.352 (0.147-0.842)*	2.840 (1.188-6.79)*	0.734 (0.416-1.297)	1.362 (0.771-2.406)	0.83 (0.457-1.504)	1.205 (0.665-2.186)
**Overweight**	0.735 (0.286-1.886)	1.259 (0.471-3.367)	1.495 (0.384-5.827)	0.669 (0.172-2.606)	0.837 (0.358-1.960)	1.194 (0.51-2.796)	0.409 (0.155-1.075)	2.448 (0.930-6.441)	0.414 (0.142-1.203)	2.417 (0.831-7.027)	0.739 (0.354-1.542)	1.353 (0.649-2.821)	1.519 (0.670-3.444)	0.658 (0.290-1.493)
**Study Fields (Reference = Health Science)**														
**Social and humunity**	0.772 (0.433-1.375)	1.304 (0.737-2.307)	0.926 (0.412-2.081)	1.080 (0.480-2.428)	1.079 (0.663-1.754)	0.927 (0.57-1.508)	0.767 (0.461-1.275)	1.304 (0.784-2.169)	0.530 (0.308-0.911)*	1.887 (1.097-3.244)*	0.973 (0.617-1.535)	1.028 (0.651-1.622)	0.811 (0.509-1.291)	1.233 (0.775-1.964)
**Sciences and Technology**	1.030 (0.558-1.901)	0.968 (0.527-1.780)	1.097 (0.471-2.556)	0.912 (0.391-2.124)	1.661 (0.984-2.803)*	0.602 (0.357-1.016)*	0.963 (0.558-1.661)	1.038 (0.602-1.791)	0.825 (0.460-1.482)	1.212 (0.675-2.176)	1.127 (0.707-1.796)	0.887 (0.557-1.414)	1.258 (0.77-2.057)	0.795 (0.486-1.299)
**Academic year (Reference = Senior)**														
**Freshman**	1.334 (0.674-2.639)	0.808 (0.412-1.582)	2.408 (0.986-5.882)	0.415 (0.170-1.014)	1.053 (0.589-1.884)	0.950 (0.531-1.698)	0.750 (0.413-1.361)	1.334 (0.735-2.422)	1.088 (0.59-2.009)	0.919 (0.498-1.696)	1.216 (0.714-2.069)	0.822 (0.483-1.400)	0.780 (0.455-1.337)	1.282 (0.748-2.197)
**Sophomore**	1.425 (0.706-2.878)	0.756 (0.379-1.508)	2.334 (0.950-5.736)	0.428 (0.174-1.053)	0.85 (0.473-1.525)	1.177 (0.656-2.113)	0.904 (0.490-1.667)	1.106 (0.600-2.041)	1.327 (0.692-2.541)	0.754 (0.393-1.444)	1.344 (0.782-2.312)	0.744 (0.433-1.279)	1.058 (0.609-1.838)	0.945 (0.544-1.643)
**Junior**	0.945 (0.496-1.803)	1.074 (0.564-2.044)	3.66 (1.423-9.414)**	0.273 (0.106-0.703)**	0.869 (0.489-1.544)	1.151 (0.648-2.045)	0.924 (0.504-1.694)	1.082 (0.590-1.982)	1.119 (0.607-2.060)	0.894 (0.485-1.646)	1.585 (0.933-2.693)	0.631 (0.371-1.071)	0.953 (0.556-1.635)	1.049 (0.612-1.798)
**Income (Reference = > 10,000 baht/month)**														
**≤5,000 baht/month**	1.645 (0.81-3.340)	0.641 (0.318-1.294)	0.874 (0.372-2.054)	0.547 (0.219-1.370)	0.739 (0.409-1.337)	1.352 (0.748-2.445)	1.048 (0.559-1.965)	0.954 (0.509-1.788)	0.701 (0.357-1.379)	1.426 (0.725-2.805)	1.824 (1.056-3.151)*	0.548 (0.317-0.947)*	0.626 (0.349-1.124)	1.598 (0.890-2.869)
**5,001-10,000 baht/month**	1.092 (0.588-2.028)	0.936 (0.506-1.732)	0.987 (0.384-2.535)	0.499 (0.213-1.166)	1.094 (0.624-1.915)	0.914 (0.522-1.602)	0.934 (0.521-1.672)	1.071 (0.598-1.919)	0.737 (0.394-1.376)	1.358 (0.727-2.537)	1.038 (0.628-1.716)	0.964 (0.583-1.594)	0.605 (0.352-1.042)	1.652 (0.960-2.844)
**Online class (Reference = > 3 days/week)**														
**≤3 days/week**	0.875 (0.536-1.430)	1.154 (0.711-1.875)	0.731 (0.371-1.441)	1.367 (0.694-2.694)	0.738 (0.488-1.116)	1.355 (0.896-2.049)	0.782 (0.507-1.208)	1.278 (0.828-1.974)	0.573 (0.362-0.907)*	1.745 (1.103-2.761)*	0.600 (0.408-0.884)**	1.666 (1.132-2.453)**	0.670 (0.451-0.996)*	1.493 (1.004-2.219)*

**Cooking method (Reference = by themself)**														
**Buying from outside**	1.868 (0.758-4.606)	0.540 (0.220-1.324)	3.544 (1.242-10.113)*	0.282 (0.099-0.805)*	0.644 (0.255-1.632)	1.552 (0.613-3.929)	3.609 (1.578-8.253)**	0.277 (0.121-0.634)**	1.680 (0.696-4.060)	0.595 (0.246-1.438)	1.527 (0.667-3.497)	0.655 (0.286-1.499)	0.753 (0.319-1.778)	1.327 (0.562-3.134)
**by parents**	2.571 (0.909-7.271)	0.402 (0.144-1.126)	3.675 (1.034-13.064)*	0.272 (0.077-0.967)*	0.824 (0.297-2.287)	1.213 (0.437-3.366)	3.195 (1.268-8.053)**	0.313 (0.124-0.789)**	2.261 (0.812-6.296)	0.442 (0.159-1.232)	2.12 (0.855-5.261)	0.472 (0.190-1.170)	0.913 (0.355-2.351)	1.095 (0.425-2.821)
**Quality of life (Reference = Poor)**														
**Mild**	1.075 (0.268-4.312)	0.938 (0.235-3.752)	12.748 (3.201-50.779)***	0.078 (0.020-0.312)***	2.871 (0.878-9.389)	0.348 (0.107-1.139)	5.914 (1.645-21.266)**	0.169 (0.047-0.608)**	5.41 (1.548-18.905)*	0.185 (0.053-0.646)*	3.440 (0.857-13.812)	0.291 (0.072-1.168)	1.462 (0.435-4.914)	0.684 (0.203-2.299)
**Good**	1.303 (0.309-5.486)	0.774 (0.185-3.235)	9.783 (2.330-41.079)**	0.102 (0.024-0.429)**	2.76 (0.817-9.32)	0.362 (0.107-1.223)	7.068 (1.900-26.298)**	0.141 (0.038-0.526)**	4.372 (1.215-15.733)*	0.229 (0.064-0.823)*	3.462 (0.843-14.225)	0.289 (0.070-1.187)	1.421 (0.411-4.909)	0.704 (0.204-2.432)

* Sig. ≤ 0.05, ** Sig. ≤ 0.01, *** Sig. ≤ 0.001
